# A Novel Quercetin Encapsulated Glucose Modified Liposome and Its Brain-Target Antioxidative Neuroprotection Effects

**DOI:** 10.3390/molecules29030607

**Published:** 2024-01-26

**Authors:** Jian Chen, Jinxia Chen, Peiyun Yu, Chunyan Yang, Chen Xia, Junlin Deng, Manyou Yu, Zuoya Xiang, Lu Gan, Boyu Zhu, Yong Wu, Xing Yang

**Affiliations:** 1 Institute of Agro-Products Processing Science and Technology, Institute of Food Nutrition and Health, Sichuan Academy of Agricultural Sciences, Chengdu 610066, China; 2 Key Laboratory of Drug-Targeting and Drug Delivery System of the Education Ministry, Department of Medicinal Chemistry, West China School of Pharmacy, Sichuan University, Chengdu 610041, China

**Keywords:** quercetin, neuroprotection, brain-target, liposome, antioxidant

## Abstract

Neurodegenerative diseases (NDDs) are mainly induced by oxidative stress which produces excessive reactive oxygen species (ROS). Quercetin (QU) is a potent antioxidant with some effects on NDDs. This study prepared and characterized a novel glucose-modified QU liposome (QU–Glu–Lip), aiming not only to overcome QU’s poor water solubility and bioavailability but also to deliver more QU to brain tissue to enhance its neuroprotective effect. QU–Glu–Lip possessed encapsulation efficiency (EE) of 89.9%, homogenous particle sizes (116–124 nm), small PDI value (<0.3), zeta value −1.363 ± 0.437 mV, proper pH and salt stability, and proper cytotoxicity. The glucose-modified liposome penetrated the blood–brain barrier (BBB) mediated via the glucose transporter 1 (GLUT1) and was taken by neuronal cells more efficiently than liposome without glucose, according to bEnd.3 and PC12 cell tests. QU–Glu–Lip attenuated H_2_O_2_-induced oxidative damage to PC12 with higher cell viability (88.42%) and lower intracellular ROS compared to that of QU. QU–Glu–Lip had higher brain target ability and delivered more QU to neuronal cells, effectively exerting the antioxidative neuroprotection effect. There is potential for the QU–Glu–Lip application for more effective treatment of NDDs.

## 1. Introduction

The World Health Organization predicts that by 2040, neurodegenerative diseases (NDDs), including cerebral ischemia, seizures, Parkinson’s disease, Alzheimer’s disease, amyotrophic lateral sclerosis, stroke, and Huntington’s disease, will replace cancer as the second most deadly disease in human beings [1,2,3]. Clinical symptoms of NDDs include memory impairment, movement perturbations, and perceptual disorders [4,5]. Oxidative stress, characterized by the excessive formation of reactive oxygen species (ROS), is one of the main pathogenic mechanisms of neurodegenerative diseases [6,7]. Based on this, the development of neuronal protective agents with antioxidant capacity is an effective strategy for the prevention and treatment of neurodegenerative diseases [8,9,10].

**Figure 1:**
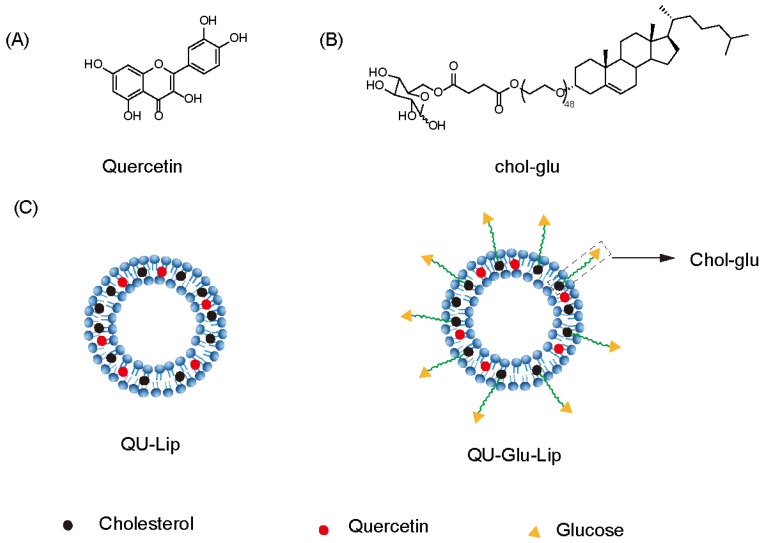
Structure of quercetin (QU), cholesterol-linked glucose ligand (Chol-glu), and liposomes. (**A**) Chemical structure of quercetin. (**B**) Chemical structure of glucose ligand. (**C**) Schematic structure of liposomes QU–Lip and QU–Glu–Lip.

Quercetin (QU, Figure 1A), a natural polyphenolic flavonoid compound (3,5,7,3′,4′-pentahydroxyflavone), is abundantly distributed among fruits, vegetables, tea, and some whole grains, nuts, and seeds [11]. It has various bioactive properties such as anti-diabetic, anti-hyperlipidemia, anti-hyperglycemia, anti-oxidant, anti-inflammatory, antibacterial, anticancer activities, and decreased blood pressure effects [12]. Recent studies suggest that the QU can exert efficacy in NDDs with neuroprotective effects [13,14,15], and the use of QU has been reported to improve pathological features in vivo NDD models such as cognitive impairment [16], ischemia, traumatic injury [17], Parkinson’s disease [18], and Huntington’s disease [19]. However, poor water solubility, instability, and low blood–brain barrier (BBB) penetration severely reduce the efficacy of quercetin and limit its clinical studies [20]. Encapsulation of QU in nanocarriers effectively improves the bioavailability of QU and allows for better efficacy [16]. For example, encapsulated QU in Eudragit-coated liposome was designed for intestinal delivery to resist the gastric acid environment [21]. The use of QU-loaded liposomes in a rat model of ischemia–reperfusion resulted in a substantial downregulation of all pro-inflammatory markers [22]. Ligand modification on the surface of liposomes also allows the liposomes to exert targeting effects. The survival of tumor-bearing mice with RGD-modified QU-loaded liposomes was significantly prolonged [23].

Penetration of the BBB is the primary challenge for the treatment of CNS diseases. The unique properties of the BBB, such as tight junctions (TJs), adherent junctions (AJs), and other junctional proteins prevent most drugs from crossing the blood–brain barrier [24,25]. Some transporters are overexpressed on the surface of BBB for transporting nutrients [26]. Glucose, an essential nutrient for the brain, is primarily transported into the brain via glucose transporter 1 (GLUT1), and with each brain capillary cell expressing about 6 × 10^6^ GLUT1 molecules [27]. Therefore, modification of the nanocarrier surface with glucose is a very effective strategy for brain targeting. In our previous studies, glucose-modified liposomes all displayed favorable brain targeting ability [28,29], but to the best of our knowledge, glucose-modified liposomal QU for brain targeting and its neuroprotective effects have not been reported.

In this study, we designed and prepared glucose-modified (via a glucose ligand, chol-glu, Figure 1B) QU-loaded liposome (QU–Glu–Lip, Figure 1C), and evaluated its BBB penetration ability and antioxidative neuroprotective effects, comparing with QU and regular QU liposome (QU-Lip, Figure 1C) via bEnd.3 and PC12 cells tests. The study provides the potential to extend the application of QU for more effective treatment of NDDs.

## 2. Results

### 2.1. Characterization of Liposomes

**Figure 2:**
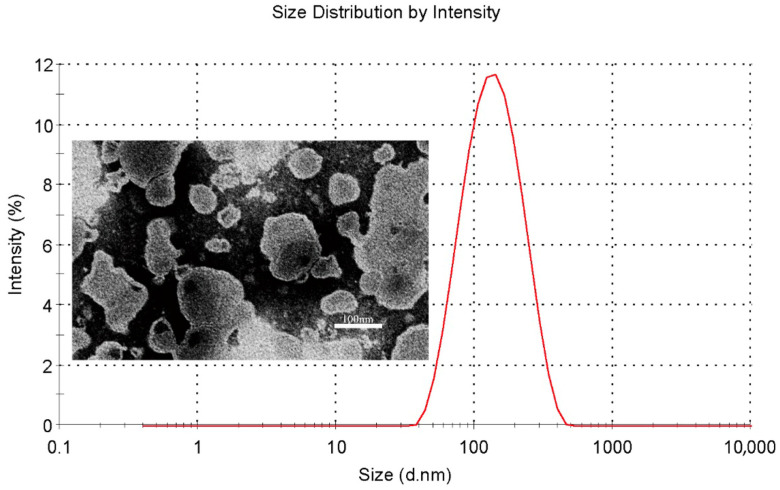
Particle size distribution (red line) of QU–Glu–Lip and its transmission electron microscopy image.

The characterization of QU–Lip and QU–Glu–Lip is shown in Table 1. Both QU–Lip and QU–Glu–Lip exhibited favorable EE (>84%) to QU. They possessed suitable particle sizes (116–124 nm) and small PDI values (<0.3), which meant they were feasible to penetrate the BBB without being phagocytosed by the reticuloendothelial system. QU–Lip had a zeta potential value of 0.348 ± 0.234 mV, but QU–Glu–Lip had a zeta value of −1.363 ± 0.437 mV. In addition, QU–Glu–Lip was a homogeneous spherical vesicle structure with a phospholipid bilayer, as shown in Figure 2.

**Figure 3:**
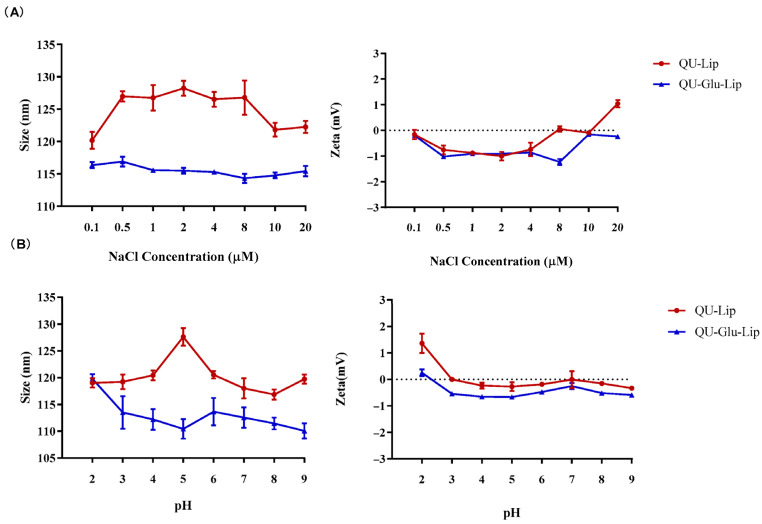
Liposomes QU–Lip and QU–Glu–Lip stabilities. (**A**) Salt stability in NaCl solution; (**B**) pH stability in PBS.

As for stability observation, no obvious changes in particle size and zeta potential were observed when QU–Lip and QU–Glu–Lip were in different concentrations of NaCl solutions and in different pH values PBS (Figure 3), indicating the comparable salt stability and pH stability of both liposomes. Furthermore, glucose-modified liposome QU–Glu–Lip was more stable than unmodified liposome QU–Lip.

### 2.2. Evaluation of the Targeting Effect In Vitro

**Figure 4:**
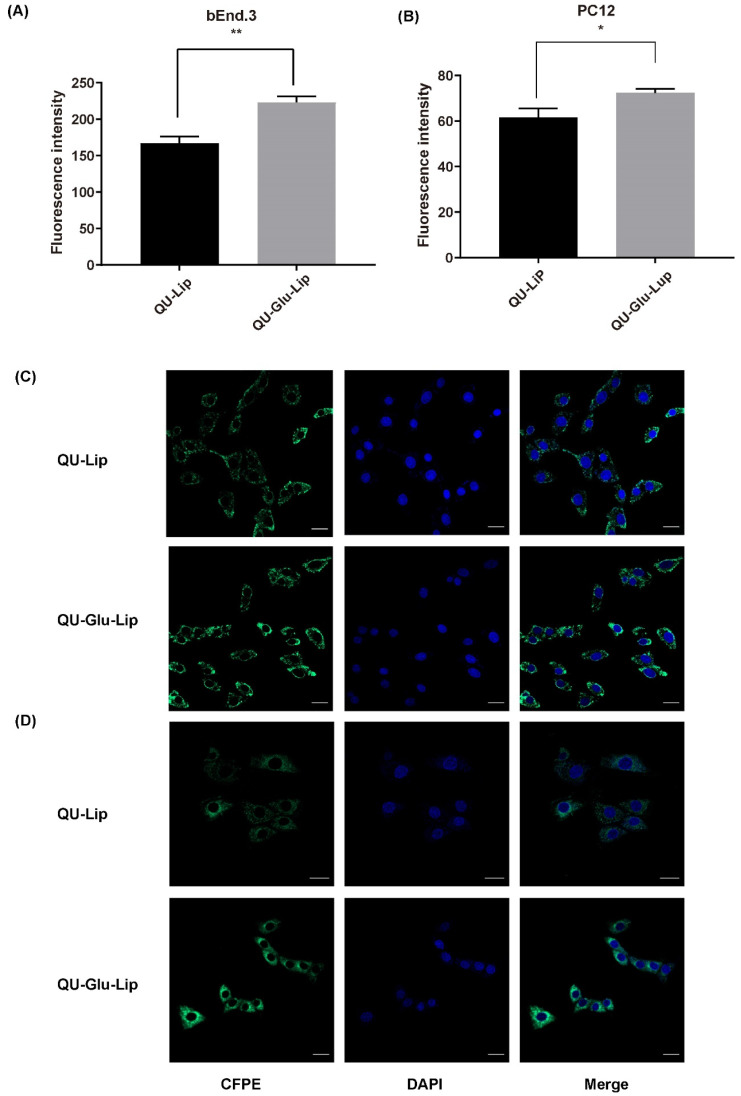
Cellular uptake of CFPE-labeled liposomes measured by flow cytometry on bEnd.3 cells (**A**) and PC12 cells (**B**); Cellular uptake of CFPE-labeled liposomes measured by confocal laser scanning microscope (CLSM) on bEnd.3 cells (**C**); PC12 cells (**D**). Green (CFPE-labeled liposomes), blue (DAPI stained nucleus), * *p* < 0.05, ** *p* < 0.01 (mean ± SD, *n* = 3, the same experiment was repeated thrice). The scale bar is 20 μm.

The targeting effect on bEnd.3 and PC12 cells was indicated by the degree of cellular uptake of liposomes. As shown in Figure 4A, CFPE–Lip had a fluorescence intensity of 167.12 on bEnd.3 cells, but CFPE–Glu–Lip was measured as high as 223.22 (*p* < 0.01). On PC12 cells (Figure 4B), CFPE–Lip’s fluorescence intensity was 61.58, while CFPE–Glu–Lip was higher at 72.41 (*p* < 0.1). The images of CFPE located in bEnd.3 and PC12 cells (Figure 4C,D) also showed that glucose modification could increase the amounts of internalized liposomes in cells. Their results (Figure 4D) showed that these liposomes were mainly located in the cytoplasm, so green fluorescence barely overlapped with the nucleus.

### 2.3. Cytotoxicity

**Figure 5:**
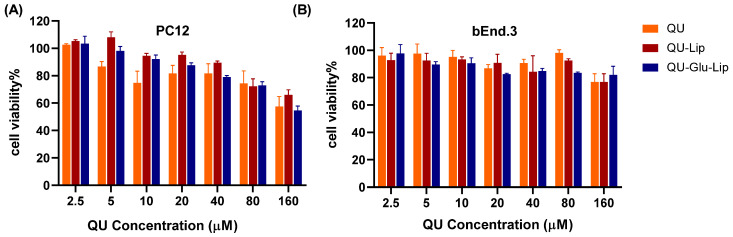
The cytotoxicity of liposomes QU–Lip and QU–Glu–Lip, and QU on PC12 cells (**A**) and on bEnd.3 cells (**B**) (mean ± SD, *n* = 3, the same experiment was repeated thrice). bEnd.3 and PC12 cells without any treatment were used as control groups (cell viability 100%), respectively.

Cytotoxicity is a key factor to be considered for neuroprotective drugs. The cytotoxicity results of QU, liposomes QU–Lip, and QU–Glu–Lip against bEnd.3 and PC12 cells are shown in Figure 5. The bEnd.3 cell viabilities of QU, QU–Lip, and QU–Glu–Lip groups were all above 80% between 2.5–80 μM concentration, and above 70% at 160 μM, indicating they were non-cytotoxicity (cell viability 80%) or weak (70%) cytotoxicity to bEnd.3 cells in the concentration range of QU at 2.5–160 μM (Figure 5B). As for PC12 (Figure 5A), the cell viabilities of the three groups were downtrended along with the increasing concentrations. All three groups’ cell viabilities were above 70% in the concentration range of QU at 2.5–80 μM, showing weak cytotoxicity to PC12 cells. But all three groups showed moderate cytotoxicity to PC12 at 160 μM, since cell viabilities of QU–Lip were below 70%, QU, and QU–Glu–Lip below 60%. Thus, 2.5–80 μM was considered the effective concentration range that could be used for their neuroprotective examinations. These results indicated that encapsulation of QU in liposomes (QU–Lip and QU–Glu–Lip) did not result in additional cytotoxicity.

### 2.4. Evaluation of Neuroprotective Effect In Vitro

#### 2.4.1. Cell Viability

**Figure 6:**
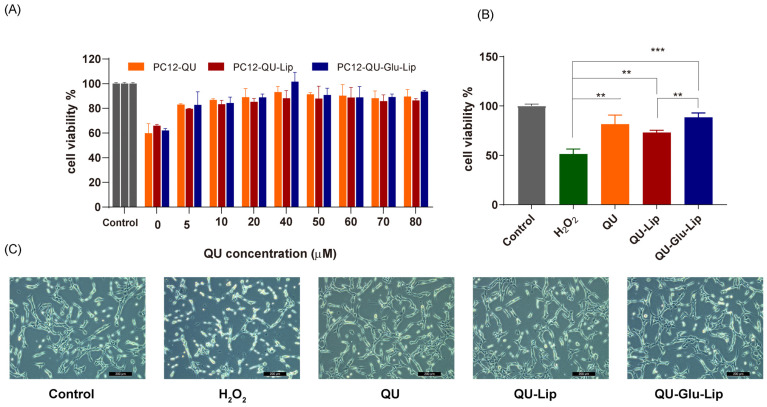
The neuroprotective effect of QU, liposomes QU–Lip, and QU–Glu–Lip on PC12 cells (PC12 cells were pretreated using 0~80 μM concentrations of QU, QU–Lip, and QU–Glu–Lip, then oxidatively damaged using 800 μM H_2_O_2_). (**A**) Cell viability comparison; (**B**) Cell viability comparison at 40 μM of QU, QU–Lip, and QU–Glu–Lip groups versus H_2_O_2_ group, ** *p* < 0.01, *** *p* < 0.001, (mean ± SD, *n* = 3, the same experiment was repeated thrice); (**C**) The morphology images on PC12 cells at 40 μM of QU, QU–Lip, and QU–Glu–Lip, scale bar 200 μm. PC12 cells without any treatment were used as the control group (cell viability 100%).

The neuroprotective effect of liposome was measured on the H_2_O_2_-induced oxidative injury PC12 cell model. According to the results shown in Figure 6A, cell viabilities were uptrend. Liposomes QU–Lip, QU–Glu–Lip, and QU all significantly increased cell viabilities with increasing concentrations in the range of 5~80 μM, compared to the cells exposed to H_2_O_2_ without pre-protection (0 μM). Since the three 40 μM groups showed the highest cell viabilities in Figure 6A, a concentration of 40 μM was chosen to further compare and evaluate the effects of QU–Lip and QU–Glu–Lip against H_2_O_2_-induced cell injury (Figure 6B). At 40 μM, the cell viability of the H_2_O_2_ group was 51.37%, the QU group was 81.58%, the QU–Lip group was 72.97%, and the QU–Glu–Lip group was the highest at 88.42%, compared with control (100%) which was not damaged by H_2_O_2_, respectively. The results showed that the viabilities of H_2_O_2_-treated PC12 cells were all increased with the pretreatment of QU (*p* < 0.01), liposomes QU–Lip (*p* < 0.01), and QU–Glu–Lip (*p* < 0.001). The results also showed that the QU–Glu–Lip group had higher cell viability than that of the QU–Lip group (*p* < 0.01), indicating QU–Glu–Lip possesses better neuroprotective ability than QU–Lip. QU–Lip is not significant to QU.

Furthermore, the cell morphology of the different groups was photographed using a microscope (Figure 6C). The cells of the control group were in good condition with clear cell boundaries, cellular fullness, transparency, and refractive properties. In the oxidatively damaged H_2_O_2_ group, the cells obviously shrank in a linear fashion, with vacuoles and increased cellular debris inside. But groups pretreated with QU, QU–Lip, and QU–Glu–Lip, followed by H_2_O_2_ oxidation, only resulted in minimal changes in cell morphology and reduced damage, with the PC12 cell condition sequence QU < QU–Lip < QU–Glu–Lip. In particular, QU–Glu–Lip treated cells showed very similar to control cells. The cell morphology also showed the stronger antioxidative neuroprotective ability of QU–Glu–Lip than QU–Lip

#### 2.4.2. Intracellular ROS

**Figure 7:**
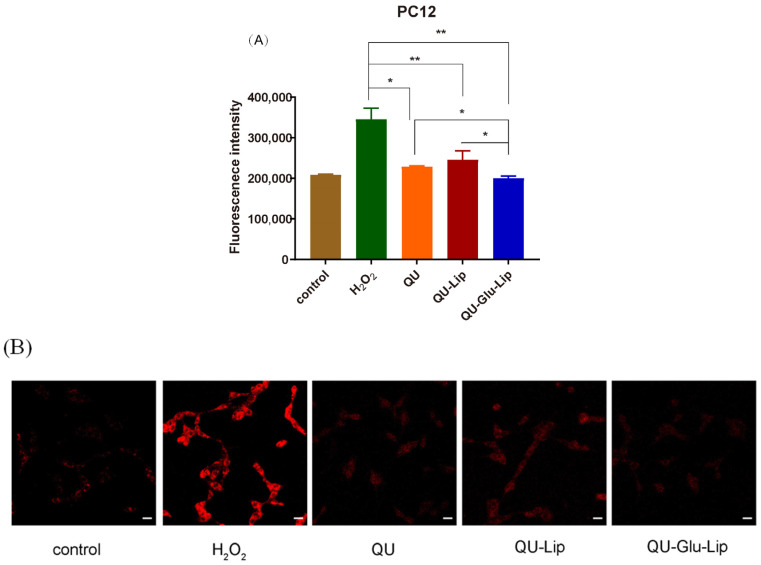
H_2_O_2_ induced ROS generation in PC12 cells pre-treated by 40 μM QU, and liposome QU–Lip and QU–Glu–Lip. (**A**) Fluorescence intensity. * *p* < 0.05, ** *p* < 0.01, versus H_2_O_2_ group (mean ± SD, *n* = 3, the same experiment was repeated thrice); (**B**) Cell image by CLSM. The scale bar is 20 μm. PC12 cells without any treatment were used as the control group.

QU has a strong antioxidant effect, which is also related to its strong scavenging ability of ROS. As shown in Figure 7A, Fluorescence intensity can indicate the amount of intracellular ROS production. In the H_2_O_2_ group, the PC12 cells oxidatively damaged by H_2_O_2_ produced large amounts of ROS, showing strong fluorescence intensity. Yet, compared to the H_2_O_2_ group, the fluorescence intensities of all the other groups were significantly lower, and in the order of groups QU–Glu–Lip > QU > QU–Lip. QU–Glu–Lip group showed significantly (*p* < 0.05) lower ROS levels than that of the QU and QU–Lip group. The result showed that QU–Glu–Lip neuroprotective ability is not only higher than QU but also higher than QU–Lip. QU–Lip is still not significant to QU.

The evaluations were simultaneously performed by qualitative observation using laser confocal microscopy (Figure 7B). The red fluorescence intensity in the figure represents the amount of intracellular ROS production. Similarly, the red fluorescence intensity was the strongest in the H_2_O_2_ group. The other groups had much weaker red fluorescence intensity, with the same QU–Glu–Lip > QU > QU–Lip sequence to fluorescence intensity. The results further demonstrated that glucose-modified QU-loaded liposome QU–Glu–Lip could more significantly reduce the intracellular ROS to reduce oxidative damage to PC12 cells.

## 3. Discussion

QU is an effective antioxidant to NDDs with neuroprotective effects, but its physicochemical properties limit its bioavailability and BBB penetration [20]. Encapsulation of QU in glucose-modified liposome getting a nanocarrier QU–Glu–Lip could improve those drawbacks. According to its characterizations, QU–Glu–Lip possessed impressive EE (89.9%) for QU delivery since liposome with EE above 70% is considered an effective drug delivery carrier. QU–Glu–Lip has a proper homogeneous particle size smaller than 200 nm for easier crossing BBB, while smaller particle sizes are more likely to be phagocytosed by the endothelial reticular system of the spleen and liver [30,31,32]. In addition, the negative charge of QU–Glu–Lip could avoid its binding to plasma proteins which possess a negative charge, thus avoiding massive accumulation in the lung and liver [33]. The salt and pH stabilities of QU–Glu–Lip also ensure that it is a reliable nanocarrier of QU.

Liposome’s selectivity of targeting neuronal cells is important to improve the neuroprotection effects of drugs. Modification of glucose ligands on the surface of liposomes could result in brain targeting through GLUT1-mediated endocytosis [28]. The brain targeting effects were evaluated using fluorescent-labeled phosphorus (CFPE) liposomes CFPE–Lip and CFPE–Glu–Lip to test their permeability on bEnd.3 cells. Some studies have shown that the degree of membrane permeability on bEnd.3 cells can indirectly indicate the penetration of BBB since bEnd.3 is a reliable BBB model [34,35]. Neuronal cell uptakes of CFPE–Lip and CFPE–Glu–Lip were tested on PC12 cells, a neuronal cell model, as the degree of membrane permeability on PC12 cells can indirectly indicate the targeting and uptake effects of neuronal cells [36,37]. The results show that the uptake of glucose-modified liposome CFPE–Glu–Lip on bEnd.3 cells was significantly 1.35 times higher than that of CFPE–Lip, implying stronger penetration ability of the BBB. On PC12 cells, CFPE–Glu–Lip was significantly 1.18 times higher than that of CFPE–Lip, implying higher uptake on neuronal cells. Since CFPE–Glu–Lip was a liposome model of QU–Glu–Lip, these results demonstrated that the glucose-modified (via Chol-glu) liposome was effective in targeting brain tissue for QU delivery.

Besides brain targeting ability, antioxidative neuroprotection effects of QU–Glu–Lip were evaluated via both cell viability and intracellular ROS on PC12 cell tests.

QU–Glu–Lip, Glu–Lip, and QU could increase PC12 cell viability, protecting cells from H_2_O_2-_induced oxidative damage. QU–Glu–lip group showed the highest cell viability, QU was in the medium, while QU–Lip showed lower cell viability. QU could enter the cell through passive diffusion to play an antioxidant effect. As for QU–Lip, there was a process of slow release of QU after the uptake of liposomes into cells, resulting in a delayed onset of antioxidant effects. The slow release of QU from QU–Lip is probably also the reason that QU–Lip group showed higher cell viabilities than QU and QU–Glu–Lip at some concentrations (Figure 5) in the cytotoxicity examinations because the slow release resulted in lower free QU concentration which induced lower cytotoxicity.

Neuroprotection effects of QU–Glu–Lip were further evaluated via intracellular ROS testing on PC12 cells. Oxidative stress caused by the accumulation of ROS is an important pathogenic factor for all NDDs [13]. It is precise to examine intracellular ROS levels to determine the neuroprotection effects of the liposome. As indicated by the results of fluorescence intensity, cells in the H_2_O_2_ group produced the highest ROS level, while the other groups’ cells produced significantly lower ROS levels in the order of QU–Glu–Lip > QU > QU–Lip. QU–Glu–Lip reduced the ROS by 1.71 times compared to the H_2_O_2_ group. In addition, the qualitative observation using laser confocal microscopy showed almost the same results. The ROS production levels in the cells of these groups were attenuated, without a big difference to that of the normal control group. The results further demonstrated that QU has an antioxidant effect, but glucose-modified liposome QU–Glu–Lip got stronger antioxidant capacity leading to a better neuroprotective effect.

## 4. Materials and Methods

### 4.1. Materials

All unspecified reagents were from commercial resources. Quercetin (QU) was obtained from Sichuan Academy of Agriculture Sciences (Chengdu, China). Soybean phospholipids (SPC, majorly with phosphatidylserine, and other components) and cholesterol (Chol) were obtained from Kelong Chemical (Chengdu, China). 4′-6-Diamidino-2-phenylindole (DAPI), 3-(4,5-dimethylthiazol-2-yl)-2,5-diphenyltetrazolium bromide (MTT), and ROS detection kit were purchased from Beyotime Institute Biotechnology (Haimen, China). 1,2-Dioleoyl-snglycero-3-phosphoethanolamine-N-(carboxyfluorescein) (CFPE) was purchased from Avanti Polar Lipids (Alabaster, AL, USA). Fetal bovine serum (FBS) was purchased from Gibico (Oakland, CA, USA). PC12 (rat adrenal pheochromocytoma cells) and bEnd.3 (mouse brain microvascular endothelial cells) cells were purchased from American Type Culture Collection. All other reagents were of analytical grade.

### 4.2. Preparation of Liposomes

The cholesterol-linked glucose ligand (chol-glu) was prepared based on our prior works [38,39], see Supplementary Materials (The synthetic rout and IR, ^1^H NMR of Chol-glu).

Glucose-modified QU-loaded liposome QU–Glu–Lip was prepared using thin film hydration sonication method [29,38]. Specifically, SPC 17.45 mg, Chol 3.89 mg, glucose ligand 2.78 mg, and QU 1.0 mg were added into a 50 mL round bottom flask, then dissolved in mixed solvent of CHCl_3_/CH_3_OH = 2/1 (*v*/*v*), and lipid film was formed by evaporated to dryness on a rotary evaporator (EYELA N-1100, Japan) under vacuum at 37 °C. The lipid film was hydrated with PBS buffer at 37 °C for 30 min and intermittently sonicated (JY92-II sonicator, Ningbo, China) at 80 W for 3 min (5 s, 5 s), centrifuged (10,000 rpm, 10 min. 3K15, Sigma, Livonia, MI, USA), and the supernatant was taken to obtain the liposome QU–Glu–Lip.

Comparative liposome QU–Lip (Figure 1C) was prepared via similar procedure, with SPC 17.45 mg, Chol 4.54 mg, and QU 1.0 mg. CFPE-labeled liposomes were prepared using same procedure substituting QU with fluorescent-labeled phosphorus (CFPE) to get CFPE–Lip and CFPE–Glu–Lip, respectively.

### 4.3. Characterization of Liposomes

The morphology of QU–Glu–Lip was imaged with transmission electron microscope (TEM, HT, Hitachi-7800, Ltd., Hitachinaka, Japan). Freshly prepared QU–Glu–Lip was diluted to 5 μmol/L with PBS (phosphate buffered saline) and added dropwise to a copper sieve, then negatively stained with 2% phosphotungstic acid (pH 4.47), after which excess staining solution was blotted with filter paper and air-dried at room temperature. Finally, transmission electron microscopy images of liposomes were captured at an accelerating voltage of 80 kV.

Freshly prepared liposomes diluted with pure water were placed in sample cuvettes, and their particle size, zeta potential, and PDI were determined with Malvern Zeta Sizer Nano ZS90 (Malvern, UK), respectively.

The encapsulation efficiency (EE) of QU in liposomes was measured using HPLC (Agilent 1200, Santa Clara, CA, USA) analysis. Each 30 µL of uncentrifuged and centrifuged liposomes was aspirated into 270 µL of methanol, respectively. The liposomes were broken up by shaking vortex (XW-80, Shanghai, China), then 20 µL of supernatant was injected into the HPLC system after centrifugation (10,000 rpm, 10 min), and detected according to the HPLC conditions in the Supplementary Materials (Chromatographic conditions and system suitability), and the EE of QU was calculated as follows:

EE(%) = A1/A2 × 100%, A1 represents the peak area of centrifuged liposomes, and A2 represents the peak area of uncentrifuged liposomes.

Liposomes salt stabilities investigation: QU–Lip and QU–Glu–Lip were co-incubated with equal volume NaCl solution at different concentrations (0.1–20 μM), respectively. The particle sizes and zeta potentials of liposomes were measured after shaking 3 h (45 rpm, 37 °C). For pH stability investigation, QU–Lip and QU–Glu–Lip were co-incubated in equal volumes of PBS at different pH values (2–9), respectively, then the particle sizes and zeta potentials of liposomes were measured after shaking 3 h (45 rpm, 37 °C).

### 4.4. Evaluation of Brain-Targeting Effect In Vitro

The bEnd.3 cells (mouse brain microvascular endothelial cells) were used as BBB model to investigate the BBB penetration effects of liposomes. PC12 cells (rat adrenal pheochromocytoma cells) were used as neuronal cell model to investigate neuronal targeting effect of liposomes. The uptake evaluation of liposomes on cells was conducted as follows:

Cells were grown in 12-well plates (3 × 10^5^) and incubated for 24 h. After incubation, cells were treated with CFPE–Glu–Lip and CFPE–Lip for 4 h under light-proof conditions, respectively, and ensured the concentration of CFPE in medium at 2 μg/mL. Afterwards, cells were collected and re-suspended in 350 μL of PBS buffer, FITC channel was selected, and the fluorescence was measured with flow cytometer (FC, BD FACS Celesta, NJ, USA).

To observe the cell image, cells were seeded in confocal image dish (5 × 10^5^) and incubated for 24 h. After that, the cells were treated with CFPE–Glu–Lip and CFPE–Lip for 4 h under light-proof conditions and ensured the concentration of CFPE in medium at 2 μg/mL. Afterward, the cells were washed with PBS, fixed with 4% paraformaldehyde, and then stained with DAPI (0.1 mg/mL) on nucleuses. Finally, the images of cells were observed with confocal laser scanning microscope (CLSM, Carl Zeiss LSM800, Oberkochen, Germany) under 60× oil magnification.

### 4.5. Evaluation of Cytotoxicity

bEnd.3 and PC12 cells were grown in 96-well plates (6 × 10^3^) and incubated for 24 h, respectively. After incubation, cells were treated with different concentrations (2.5, 5, 10, 20, 40, 80, 160 μmol/mL) of QU–Glu–Lip, QU–Lip, and free QU for another 24 h, respectively. The cell viability was evaluated via MTT assay following the standard process. bEnd.3 and PC12 cells without any treatment were used as control groups (cell viability 100%), respectively.

### 4.6. Evaluation of Neuroprotective Effect In Vitro

The neurotoxicity model in vitro was established by inducing oxidative stress on PC12 cells with H_2_O_2_ in 800 μM. The neuroprotective effect of liposomes was assessed by measuring the cell viability and ROS levels.

PC12 cells were grown in 96-well plates (1 × 10^4^) and incubated for 24 h. After incubation, cells were treated with different concentrations (0, 5, 10, 20, 40, 50, 60, 70, 80 μM) of QU–Glu–Lip, QU–Lip, and QU for 2.5 h, respectively, and then exposed to H_2_O_2_ for 2 h. PC12 cells without any treatment were used as control group (cell viability 100%). The cell viability was evaluated via MTT assay, and the cell image was observed by microscopy.

To investigate the intracellular ROS generation, PC12 cells were seeded in six-well plates (6 × 10^5^) and incubated for 24 h. After incubation, cells were treated with QU–Gl–Lip, QU–Lip, and QU in 40 μM for 2.5 h, respectively, and then exposed to H_2_O_2_ for 2 h. Next, cells un-harvested were stained with 2′,7′-dichlorofluorescein diacetate (DCFH-DA, 10 μM) and incubated for 30 min in an incubator in the dark. Afterwards, cells were collected and re-suspended in 350 μL of PBS buffer, FITC channel was selected, and the fluorescence was measured with flow cytometer (FC, BD FACS Celesta, San Francisco, CA, USA). PC12 cells without any treatment were used as control group.

To observe the ROS fluorescence intensity, cells were seeded in confocal image dish (5 × 10^5^) and incubated for 24 h. Next, cells were stained with DCFH-DA (10 μM) and incubated for 30 min in an incubator in the dark. Finally, the cells were fixed with 4% paraformaldehyde and placed under a confocal laser scanning microscope (CLSM, Carl Zeiss LSM800, Oberkochen, Germany) to observe the fluorescence intensity through the DCF channel. PC12 cells without any treatment were used as control group.

### 4.7. Statistical Analysis

Statistical data processing was performed using GraphPad Prism 8.0 software and statistical comparisons of multiple groups were performed by one-way or two-way ANOVA. Differences were considered significant at *p* < 0.05. Results are presented as mean ± standard deviation (M ± SD).

## 5. Conclusions

A novel liposome QU–Glu–Lip was prepared and encapsulated sufficient QU, with glucose modified on the nanocarrier surface. QU–Glu–Lip possessed proper salt stability and pH stability, homogeneous particle size, negative charge, and proper cytotoxicity to neuronal cells. These characteristics enable QU–Glu–Lip to penetrate BBB mediated via GLUT1, leading to increased delivery and more precise brain targeting of QU in brain tissue. The glucose-modified liposome could further carry more QU into neuronal cells. As evaluated via cell viability and ROS level of examinations, QU–Glu–Lip exerted more effective antioxidative neuroprotective effects than QU. There is potential to further study QU–Glu–Lip for its effective application in NDDs.

## Figures and Tables

**Table 1:** Particle sizes, PDI, EE, and zeta potentials of QU-loaded liposomes (mean ± SD, *n* = 3, the same experiment was repeated thrice).

Liposomes	Size (nm)	PDI	EE (%)	Zeta Potential (mV)
QU–Lip	123.4 ± 0.322	0.234 ± 0.012	84.31 ± 0.887	0.348 ± 0.234
QU–Glu–Lip	116.1 ± 0.973	0.212 ± 0.002	89.9 ± 1.752	−1.363 ± 0.437

## Data Availability

Data are contained within the article or Supplementary Material.

## Supplementary Materials

The following supporting information can be downloaded at: https://www.mdpi.com/article/10.3390/molecules29030607/s1, Section S1: The synthetic rout and IR, ^1^H NMR of Chol-glu; Section S2: Chromatographic conditions and system suitability.
