# Supplementary material for: A Novel Quercetin Encapsulated Glucose Modified Liposome and Its Brain-Target Antioxidative Neuroprotection Effects
Source: Molecules. 2024 Jan 26;29(3):607. doi: 10.3390/molecules29030607 (PMC10856503; doi:10.3390/molecules29030607)
Supplement: Supplementary file 1 [file molecules-29-00607-s001.zip › molecules-2743449-supplementary.pdf]

Supplementary materials

S1. The synthetic route and IR, ^{1}H NMR of Chol-glu

The synthesis of **Chol-glu** is showed in **Scheme S1**. The C1,2,3,4,6-OH of glucose was protected by TMS to give 1,2,3,4,6-penta-O-(trimethylsilyl)-D-glucopyranose. Then the C6-OH was selectively deprotected and condensed with monobenzyl succinate, and finally hydrogenated to remove the benzyl group to give 1,2,3,4-tetra-O-(trimethylsilyl)-6-succinyl-D-glucopyranose. For the cholesterol portion, the 3β-OH configuration was first flipped to the 3α configuration, then etherified with triethylene glycol, and the terminal hydroxyl group was reacted with p-toluenesulfonyl chloride to form a sulfonate ester (Compound 4), and then etherified with PEG2000/NaH to obtain Compound 5, which was then reacted with the above synthesized 1,2,3,4-Tetra-O-trimethylsilyl-6-butanedioyl-D-glucose, and finally the glucose protecting group was removed to obtain **Chol-glu**. IR (KBr): 3471, 2971, 2934, 1750, 1249, 1112 cm^{-1}, ^{1}H NMR (400 MHz, CDCl3 , δ ppm): 5.24 (m, 1H, chol H-6), 5.20 (m, 1H, H-1), 4.91 (m, 1H, H-6), 4.50 (m, 1H, H-2), 4.21 (m, 1H, H-5), 4.15 (m, 1H, H-4), 3.74–3.36 (m, PEG2000-H and 4 × CH_2CH_2O), 3.33 (m, 1H, H-3), 3.28 (m, 1H, H-6′), 3.08 (m, 1H, chol H-3), 2.57 (m, 4H, $COCH_2CH_2CO$), 2.28–0.58 (remaining chol protons) with 0.58 (s, 3H, CH_3 -18), 0.76 (d, 6H, CH_3 -26, CH_3 -27, J = 6.7 Hz), 0.81 (d, 3H, CH_3 -21, J = 6.8 Hz), 0.93 (s, 3H, CH_3 -19).

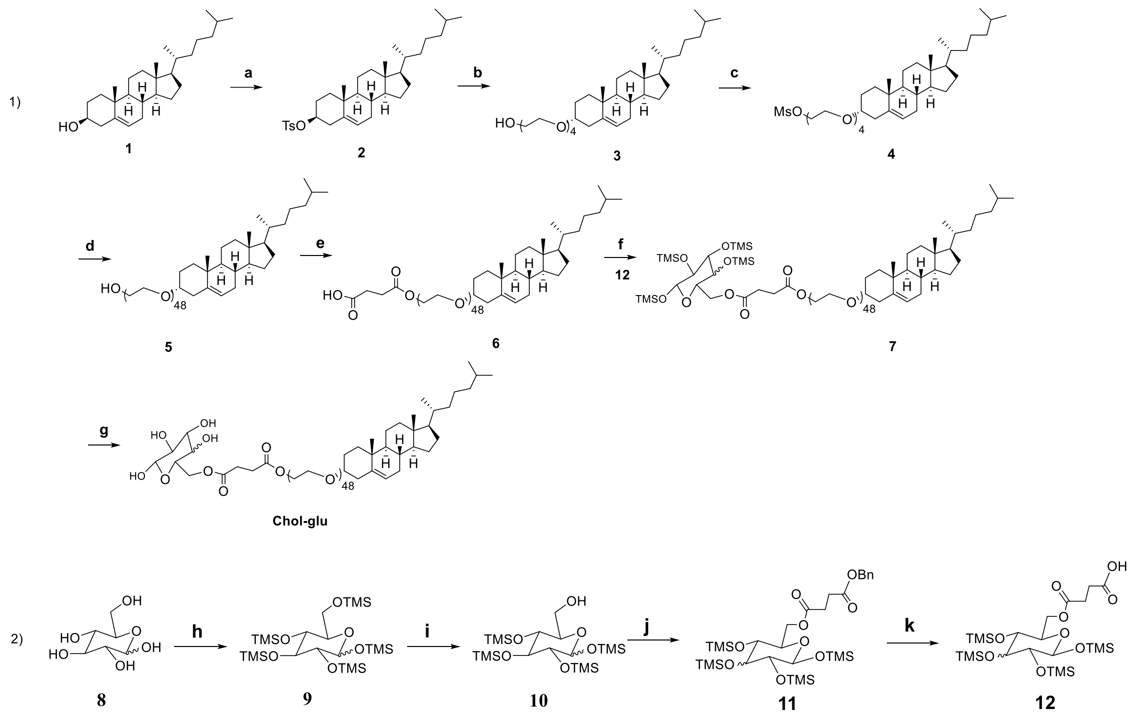

Scheme S1. The synthetic route of **Chol-glu**. Reagents and conditions: (a) TsCl, prydine, 50°C, 8 h; (b) $HOCH_2(CH_2OCH_2)_2CH_2OH$, dioxane, reflux, 8 h; (c) MsCl, Et_3N, THF, reflux, overnight; (d) PEG2000, NaH, THF, r.t., reflux, 20 h; (e) succinic, anhydride, pyridine, 75°C, 8 h; (f) Compound 12, DCC, DMAP, CH_2Cl_2, -10°C, r.t., 24 h; (g) CF_3COOH, r.t., 4 h. (h) HMDS, TMSCl, Pyridine, 0°C, r.t., 24 h; (i) AcOH, CH_3OH, acetone, 0°C, r.t., 2 h; (j) DCC, DMAP, CH_2Cl_2, r.t., 4 h; (k) Pd/C (10%), H_2, CH_3OH, r.t., 2 h.

S2. Chromatographic conditions and system suitability

The regression equation between the injection volume and peak area under chromatographic conditions was y=22.76x-19.8, R^2=0.9999 (x = quercetin concentration, y = quercetin peak area). The results in **Fig. S2** show that there is a good linear relationship between the quercetin injection volume and the peak area within the concentration range of 2.5 μg/L to 100 μg/L. The liposome membrane materials were examined under the chromatographic conditions in which the liposome materials did not affect the quercetin peak, as shown in Fig. S2. Quercetin alone peaked at 6.12 min with good peak shape as shown in Fig. S2 (B). Fig. S2 (A) showed the chromatogram of the solvent alone (mobile phase) under the above

chromatographic conditions. The solvent did not affect the peak shape of quercetin as there were no other peaks present at the time of quercetin peak. Fig. S2 (C) showed the chromatogram of the blank liposome Lip after vortex fragmentation in methanol under these chromatographic conditions the lipid material has no effect on the quercetin peak. Figure S2 (D) showed the chromatogram of QU-Glu-Lip after vortex fragmentation in methanol with good quercetin peak shape, indicating that this chromatographic condition can be used to determine the amount of quercetin encapsulated in the QU-Glu-Lip.

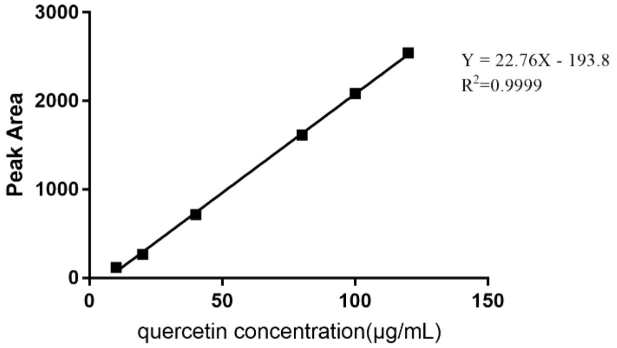

Fig.S2 Quercetin concentration versus peak area linearity

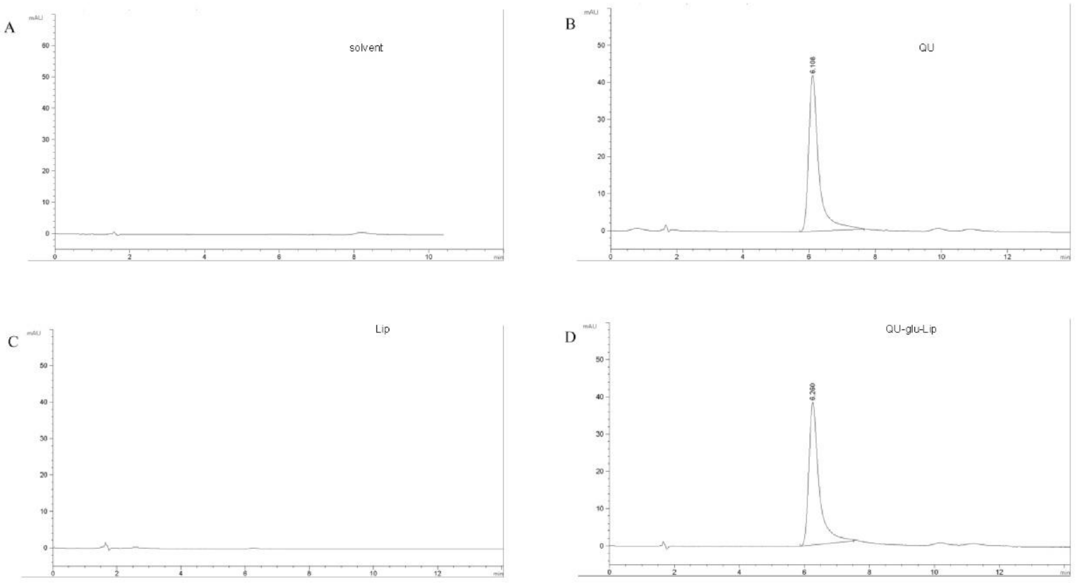

Fig.S2 The solvent, quercetin, and liposomes chromatograms
